# An Image Fusion Method of SAR and Multispectral Images Based on Non-Subsampled Shearlet Transform and Activity Measure

**DOI:** 10.3390/s22187055

**Published:** 2022-09-18

**Authors:** Dengshan Huang, Yulin Tang, Qisheng Wang

**Affiliations:** College of Civil Engineering, Xiangtan University, Xiangtan 411105, China

**Keywords:** remote sensing, image fusion, SAR, multispectral images, shearlet transform, activity measure

## Abstract

Synthetic aperture radar (SAR) is an important remote sensing sensor whose application is becoming more and more extensive. Compared with traditional optical sensors, it is not easy to be disturbed by the external environment and has a strong penetration. Limited by its working principles, SAR images are not easily interpreted, and fusing SAR images with optical multispectral images is a good solution to improve the interpretability of SAR images. This paper presents a novel image fusion method based on non-subsampled shearlet transform and activity measure to fuse SAR images with multispectral images, whose aim is to improve the interpretation ability of SAR images easily obtained at any time, rather than producing a fused image containing more information, which is the pursuit of previous fusion methods. Three different sensors, together with different working frequencies, polarization modes and spatial resolution SAR datasets, are used to evaluate the proposed method. Both visual evaluation and statistical analysis are performed, the results show that satisfactory fusion results are achieved through the proposed method and the interpretation ability of SAR images is effectively improved compared with the previous methods.

## 1. Introduction

In the past decades, with the rapid development of remote sensing technology, multiple types of remote sensing satellites have been sent into outer space, i.e., multispectral, hyper-spectral and synthetic aperture radar satellites [1]. The data from these various satellites is used for different purposes, such as national defense, agriculture, land use/land cover, geology, urban change detection and so on [2]. However, owing to some technical constrains and defects of their imaging mechanism, the information captured by sensors alone is limited, which cannot meet the needs of many practical applications completely. Optical sensors and SARs are typical representatives in this field. Optical satellite data is most suitable for human perception, which has been widely used in practice. However, as the storage space and the electromagnetic wave bandwidth of sensors used to communicate with ground receiving stations are limited, it is difficult for an optical sensor to capture images with both a high spatial resolution and a high spectral resolution simultaneously, thus many sensors are designed to have a panchromatic band with a high spatial resolution and several multispectral bands with a low spatial resolution, such as Landsat, IKONOS and so on. Optical sensor imaging has a serious defect, that is, it is easily affected by external environment, such as rain, snow, clouds and night, etc., which seriously limit the use of optical satellites in practice [3]. Compared with optical remote sensing sensors, SARs are active remote sensing sensors that emit microwaves by themselves, which could not be affected by weather change or imaging time, and the earth surface can be observed under the conditions including rain, snow, clouds and even night thanks to their capability. Due to the long wavelength of SARs, they even can penetrate some materials on the earth surface and obtain information of subsurface features [2,4]. The information contained in SAR images is mainly the moisture, roughness and terrain of the observed surface. In addition, some materials will be clearly reflected in the images, such as metal materials. All these characteristics make SAR images completely different from visual light images, the information they contain will be a complement for visual light data. However, SAR data lacks spectral information due to the single frequency used, making it quite difficult for people to interpret SAR images correctly [5]. In order to make full use of the potential of SAR data and better understand the research area to meet practical work needs, it is necessary to fuse SAR and optical images from different sensors to generate a fused image, which is more informational and reliable as a feasible method [6,7]. The specific goal is to inject spectral information of multispectral images into SAR images as much as possible to improve the interpretation ability of SAR images.

In general, image fusion can be simply defined as “the combination of two or more different images to produce a new image with a greater quality and reliability using a certain algorithm” [7,8,9]. At present, many algorithms have been proposed to address the problem of the fusion of SAR and optical images [1,7,10]. According to the published literatures, the main fusion methods of SAR and optical images can be roughly classified into four categories: component substitution (CS) methods, multi-resolution decomposition methods, hybrid methods and model-based methods [1,11]. The principle of component substitution methods is that multispectral images are projected into another space to separate spatial and spectral information, then spatial components are substituted by SAR images, subsequently, an inverse transformation is performed on the optical images projected into the original space and the image fusion task is finished. Intensity-Hue-Saturation transform (IHS), principal component analysis (PCA) and Gram–Schmidt transform (GS) are the main methods belonging to this category [12,13]. The structure of this type of method is relatively simple and the amount of calculation is small, so they have been integrated into some commercial remote sensing image processing software, e.g., ENVI and ERDAS. However, fused images often contain serious spectral distortion owing to large differences between SAR images and optical images. Multi-resolution analysis (MRA) methods mainly include pyramid decomposition, discrete wavelet transform (DWT), curvelet transform and contourlet transform [14,15,16], etc. Source images to be fused are first decomposed into several scale levels, then fusion tasks are performed on source images of each level according to specific fusion rules, and finally a reverse transform is used to produce fused images. Through this type of method, spectral distortion can be reduced, and signals can be increased to noise ratio at the cost of a large count computational complexity. The hybrid methods of IHS transform and MRA are presented to overcome the shortcomings of CS and MRA methods mentioned above. Chibani et al. made use of A’trous wavelet to extract the features of SAR images, and integrate them into multi-spectral images through IHS transform. Hong et al. proposed a high-resolution method for the fusion of SAR and moderate-resolution multispectral images based on IHS through discrete wavelet transform [4]. Chu et al. presented a method to merge PalSAR-2 SAR and Sentinel-2A optical images using IHS and shearlet transform [15]. Recently, fusion methods based on models are also used to merge SAR and optical images such as variational models, sparse representation as well as deep learning [17,18,19], etc. The main idea of this type of fusion method based on variational models is that the difference between the pixel intensity of fused images and source images should be as small as possible, thus the image fusion problem is converted into a problem of objective function optimization [17]. Shao et al. expanded this idea to fuse SAR and multispectral images based on the minimal gradient and intensity difference between the fused images and source images [20]. An over-complete dictionary was first created based on some related images through the fusion method using sparse representation; secondly, source images were represented according to the dictionary, and then the coefficients were calculated according to fusion rules; finally, fusion result was constructed based on the dictionary. The key question of the above fusion methods is how to create a dictionary through this approach. At present, deep learning is a hot point in the image processing field, which is also used for remote sensing image fusion. In the fusion method proposed by Shakya et al., deep learning was used to determinate the weight in the fusion process through the learning of specific images [18]. However, it can be found from the algorithm structure that this type of method is relatively complex and requires a lot of calculation. Meanwhile, the amount of remote sensing image data is usually huge, thus the hybrid methods are more suitable for remote sensing image fusion at present.

During the image fusion process using a hybrid model, MRA tools and fusion rules are two main problems. The MRA tools could be used to decompose images, which include pyramid decomposition, wavelet transform, curvelet transform, contourlet transform and shearlet transform, etc. Compared with other MRA tools, the shearlet transform has the best capability to describe the detailed image information [21]. If the subbands of the images are better separated, the images are more easily injected into relevant information, and fusion rules determine the purpose as well as evaluation criteria of image fusion. Chibani et al. injected the texture information of SAR images into multispectral images. Alparone et al. extracted texture information of SARs using A’trous wavelet and added the information to multispectral images [22]. Youcef et al. utilized A’trous wavelet to decompose SAR images, then integrated high-frequency components of them to the intensity components of multispectral images [14]. All these fusion rules mentioned above indicate that their aim is to generate an improved multispectral image. However, in practice, SAR images are easier to be obtained than optical images, especially in the case of bad weather at some places, thus SAR images can reflect the surface conditions more quickly. Meanwhile, it is difficult to capture optical images. So, what we really need is to enhance the interpretability of SAR images in such conditions, rather than improving multispectral image information. Hong et al. proposed a method based on IHS transform and wavelet decomposition to merge SAR images with multispectral ones. Firstly, multispectral images were converted into IHS space, then SAR images and the intensity components of multispectral images were decomposed, new low-frequency components were replaced through a weighted combination of both SAR and optical images, while the high-frequency components of SAR images were adopted to the new high-frequency components, an inverse wavelet transform was performed to obtain a new intensity, finally, an inverse IHS transform was used to finish the whole fusion process, during which SAR image information was retained completely. Meanwhile the color information of multispectral images was fused into new images, which would greatly help to improve the interpretation ability of SAR images [4]. However, due to the weak ability of wavelet transform to depict image details, there is still space for the improvement of the fusion effect. Chu et al. [15] proposed a fusion method using shearlet and IHS transform, whose process was similar to Hong’s method, and the difference lay in the image decomposition methods and fusion rules. Compared with wavelet transform, more spatial details of the images can be captured through shearlet transform. It is proposed to determine the weight of low-frequency component coefficients and use the difference of a coefficient variation of a 3 × 3-size window. The high-frequency coefficients are selected from the high-frequency coefficients of SAR and optical images according to the criterion of the maximum energy in a 3 × 3-size window, whose advantage is that the information changed in SAR and optical images will be integrated into the new images, and the information of the new images will be richer. The disadvantage is that the fusion rules may destroy the original structure of spatial information and bring difficulties to the interpretation of fused images. Sheng et al. [23] also proposed a similar method, a fusion rule using structure similarity and luminance difference of sparse representation in a low-frequency domain, and a sum-modified-Laplacian pyramid in a high-frequency domain was used. Good fusion results were obtained through the method with little color distortion. However, owing to the requirement of building an over-complete dictionary, it needs a lot of computation, and some SAR image information would be lost, which might cause some troubles for SAR image interpretation.

Keeping this in view, in order to make better use of the SAR images in which ground spatial information can be collected without being affected by external conditions and increase the interpretability of the SAR images, a novel fusion method of SAR and multispectral images is proposed in this paper based on shearlet and activity measure. The goal of image fusion is to inject the color and spatial information contained in multispectral images into SAR images as much as possible, so as to improve the interpretability of the SAR images. The main steps are similar with those of Shearlet+IHS fusion method [15]. The difference between the methods mainly lies in the difference of their fusion purpose and fusion rules. In order to verify and evaluate the method proposed in this paper, three datasets of different remote sensing images are used for fusion and evaluation. One dataset contains Sentinel 1A images and Sentinel 2B images, one contains ALOS PALSAR images and landsat5 TM images, and the other contains GF3 images and Sentinel 2A images. In addition, considering that different polarization modes of SARs have a great impact on the strength of SAR signals, each dataset in this paper includes co-polarization SAR images and cross polarization SAR images. Fusion results are evaluated visually and statistically, which are compared with those of other methods, such as IHS, DWT+IHS and Countourlet+IHS, Fused images of different polarization modes are also compared. Visual and statistical evaluations demonstrate that better fusion results could be obtained and produced through the method proposed in this paper compared with other methods, successfully injecting the spectral and spatial information of multispectral images into SAR images, and the amount of information as well as interpretation ability of SAR images is greatly improved.

The rest of the paper is organized as follows. The experiment data and the fusion method proposed in this paper are described in detail in Section 2. Then Section 3 provides experiment results and analyses. The experiment results are discussed in Section 4 and the paper is concluded finally in Section 5.

## 2. Materials and Methods

### 2.1. Data

Three datasets from different sensors are used to evaluate the validity and effectiveness of the fusion method proposed in this paper. The detailed spatial resolutions and electromagnetic spectral ranges of the image data are listed in Table 1.

The first dataset includes Sentinel 1A SAR and Sentinel 2A multispectral images, which were captured on 16 and 14 May 2016. The scenes lay in Xiangtan City, Hunan Province, Central South China. Geographical coordinate range is from 27°47′43″ N to 27°53′21″ N and from 112°48′58″ E to 112°55′07″ E. The image size is 512 × 512 pixels (actual area is 5.1 km × 5.1 km). The band of Sentinel 1A SAR is Band C, the polarization models of Sentinel 1A images are Vertical transmit and Vertical receive (VV), Vertical transmit and Horizontal receive (VH), and their spatial resolution is 15 m. The second dataset includes ALOS PALSAR images and lansat5 TM multispectral images, which were captured on 17 and 12 December 2010. The scenes lay in Xiangtan City, Hunan Province, Central South China. Geographical coordinate range is from 27°49′15″ N to 27°57′03″ N and from 112°49′09″ E to 112°58′12″ E. The image size is 512 × 512 pixels (actual area is 7.3 km × 7.3 km). The electromagnetic band of PAL SAR images belongs to Band L. The polarization models of PAL SAR images are Horizontal transmit and Horizontal receive (HH), Horizontal transmit and Vertical receive (HV), whose spatial resolution is 12.5 m. The third image dataset contains GF3 SAR images and Sentinel2A optical images obtained on 11 June and 15 July 2021 respectively. The study area is located in the north of Zhengzhou City, Henan Province, China. Geographical coordinate range is from 34°53′37″ N to 34°58′48″ N and from 113°56′13″ E to 114°01′05″ E. The image size is 512 × 512 pixels (actual area is 4.3 km × 4.3 km). The electromagnetic band of GF3 SAR images belongs to Band C, the polarization models of GF3 SAR images include HH and HV, and the spatial resolution is 8.5 m. The multispectral images used in this paper include Sentinel 2A images and Landsat 5 TM images. The electromagnetic bands of optical image include red, green, blue and near-infrared bands. Combinations of red, green, blue and near-infrared bands, red and green ones are used in this paper to finish image fusion. The combination of red, green and blue bands shows the true color images, which are most suitable for human visual perception. The combination of near-infrared red and green bands can highlight vegetation and water. The study area Zhengzhou is located in the north of China, where the main terrain is plain. Xiangtan is located in the south of China, where the main terrain is hilly and covered with vegetation. The study area surface coverings mainly include water body, vegetation, bare lands and residential areas, etc.

### 2.2. Data Preprocessing

Before the fusion of SAR images and optical images, data preprocessing processes such as noise removal, terrain correction and image registration are also needed. Due to the influence of radar signal interferences, SAR images show a lot of random speckle noise, which seriously affects the image interpretation. The noise reduction of SAR images is divided into two steps. The first is multi-look step, namely averaging the spatial resolution in range and azimuth to suppress the speckle noise contained in SAR images. Secondly, a filter is carried out. SAR image filtering is also a hot issue of SAR image processing. The main methods include Lee, Enhanced Lee, Frost, Enhanced Frost and Gamma MAP [24,25], etc. After comparing the filtering effect, an enhanced Frost filter is selected (window size 5 × 5) and applied to reduce speckle noise in this paper. Then, the SAR images need to be corrected based on a digital elevation model (DEM) to eliminate the geometric deformation caused by terrain change, and DEM SRTM3 is used in this process. Finally, the images are registered with each other. Generally, the images with a high spatial resolution are used as the reference image, and those with a low spatial resolution are the registered images. World Geodetic System Coordinate System (WGS84) is adopted for all data, and the projection model is Universal Transverse Mercator Projection (UTM). The software of Sentinel Application Platform (SNAP) is used for image preprocessing.

### 2.3. Method

#### 2.3.1. IHS Transform

IHS color space is the most suitable color description for human visual perception. Hue represents different colors, saturation refers to their purity, and intensity represents their brightness. It can be seen from the definition mentioned above that the color information of IHS color space is mainly concentrated in components H and S. However, RGB color space is suitable for color description on computer, in which the principle of additive method is applied to produce various colors. Therefore, it is widely used to convert remote sensing images from RGB space to IHS space for image fusion. IHS method has advantages including a simple structure and a small amount of calculation, which is very suitable for the processing of large amount of data such as remote sensing images. But its shortcomings are also very prominent, e.g., it will cause obvious spectral distortion in the fusion process, and can only deal with three different bands at the same time. IHS transformation forms include linear and nonlinear transformation. The specific formula of linear transformation is described as follow [26]:

(1) $$\left(\begin{array}{c} I\\ v1\\ v2 \end{array}\right)=\left(\begin{array}{ccc} \frac{1}{3} & \frac{1}{3} & \frac{1}{3}\\ -\frac{\sqrt{2}}{6} & -\frac{\sqrt{2}}{6} & \frac{2\sqrt{2}}{6}\\ \frac{1}{\sqrt{2}} & -\frac{1}{\sqrt{2}} & 0 \end{array}\right)\left(\begin{array}{c} R\\ G\\ B \end{array}\right)$$

where

$$H=\tan^{-1}(\frac{v2}{v1})\;S=\sqrt{v1^{2}+v2^{2}}$$

And the corresponding inverse transformation is:

(2) $$\left(\begin{array}{c} R\\ G\\ B \end{array}\right)=\left(\begin{array}{ccc} 1 & -\frac{1}{\sqrt{2}} & \frac{1}{\sqrt{2}}\\ 1 & -\frac{1}{\sqrt{2}} & -\frac{1}{\sqrt{2}}\\ 1 & \sqrt{2} & 0 \end{array}\right)\left(\begin{array}{c} I\\ v1\\ v2 \end{array}\right)$$

#### 2.3.2. Non-Subsample Shearlet Transform

**Figure 1:**
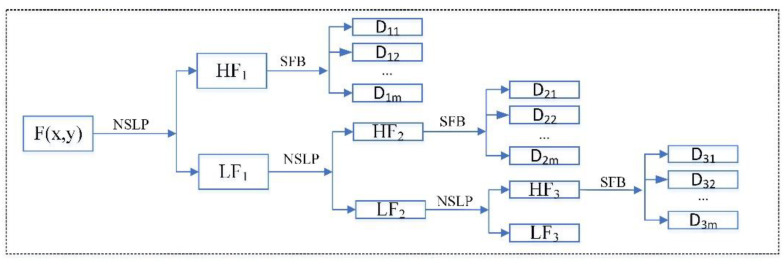
Diagram of image non-subsampled shearlet decomposition.

Shearlet transform is a new multi-resolution analysis tool after the wavelet transform, curvelet transform and contourlet transform proposed in the field of digital image processing. After images are decomposed using wavelet transform, three components will be generated in the horizontal, vertical and diagonal direction of the high-frequency domain. Due to the limited directions, it is difficult to accurately describe the edge information of spatial objects in different directions, which will thus lead to a poor detail injection effect during image fusion proceeding. Moreover, in the process of signal wavelet transform, down-sampling is also required after the signals are filtered, so that the wavelet transform no longer has the characteristics of translation invariance, which will result in the pseudo-Gibbs phenomenon in the reconstructed images, and the specific performance is that irregular edges as well as ghosts easily appear on the fused images. Curvelet transform and contourlet transform, proposed subsequently, make up for the defect of wavelet transform in representing image multi-directional features in a great measure, but the image multi-resolution decomposition process does not have the characteristics of translation invariance, which will also cause the pseudo-Gibbs effect in the reconstruction of decomposed images. This defect is overcome in a great measure through the proposal of non-subsampled contourlet transform (NSCT). However, this method has the problem of a high computational complexity and a long computational time. The non-subsampled shearlet transform (NSST) proposed by Easley et al. [21,27] is a new multi-resolution decomposition algorithm, which has the characteristics of translation invariance in the process of image multi-resolution decomposition, and each decomposed level has multiple different directions. The algorithm has a high operation speed, which has been applied to various types of image fusion and achieved good fusion results [15,28]. NSST transform mainly includes two steps: multi-resolution decomposition and multi-directional decomposition. Multi-resolution decomposition is to filter the images using non-subsampled Laplacian pyramid filter bank (NSLP). After n times of filtering, the source image is decomposed into one low-frequency approximate image and n high-frequency detail images. The size of high-frequency and low-frequency images is the same as that of the source image. Then, a shearlet filter bank (SFB) is used to decompose high-frequency images into multiple-direction images through multidirectional decomposition processing. Through ingenious system mapping and Fourier transform, the down sampling operation is avoided in the process of shear wave filtering and the translation invariance of information transformation is realized. As is shown in Figure 1, an image is decomposed three times using the non-subsampled shearlet transform, and the high-frequency components (HF) of each level are decomposed into m directions.

#### 2.3.3. The Proposed Fusion Method

**Figure 2:**
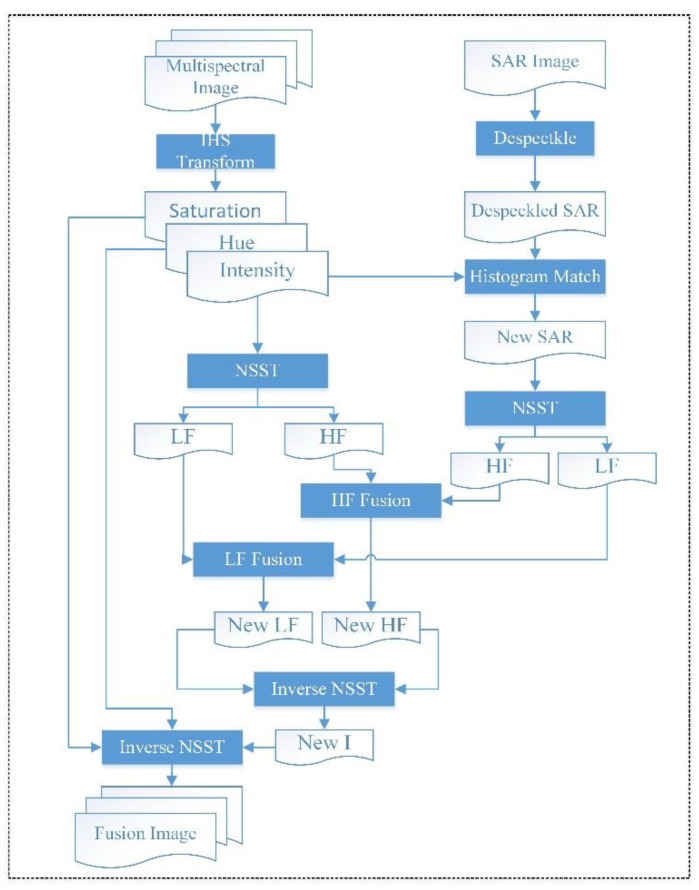
The flowchart of the image fusion method proposed.

A method for the fusion of SAR and optical images based on non-subsampled shearlet transform and activity measure is proposed to improve the interpretation ability of SAR images in this paper. The specific purpose is to integrate the spectral information and detailed information of optical images into SAR images as much as possible while keeping the SAR image information basically unchanged. It is expected that the fused images contain the detailed spatial information of SAR images and the spectral information of multispectral images, so that we can better interpret SAR images with the help of optical image information. The NSST toolkit used in this paper are provided by literature [27], which can be obtained from link https://github.com/WeiTan1992/NSST-MSMGPCNN/tree/master/nsst_toolbox, accessed on 5 March 2022. The image decomposition parameter is 5, the corresponding direction number of each layer is 0, 8, 8, 16, and 16 in this paper. The detailed fusion steps are shown in Figure 2.

1. Multi-spectral images are transformed into IHS space using Equation (1).
2. To avoid huge gray value distribution difference, the histogram of SAR images is matched to that of the intensity components.
3. Intensity components and SAR images are decomposed using non-subsampled shearlet transform, whose decomposition level is 3.
4. A weighted combination is performed between the low-frequency components of SAR images and Component I to generate new low-frequency components. The weight is determined by Equations (3)–(5) below.
5. The activity measure for high-frequency components of both SAR images and intensity components is computed according to Equations (6) and (7), and a new high-frequency component is produced according to Equation (8).
6. An inverse shearlet transform is performed to produce a new intensity.
7. An inverse IHS transform is conducted using new intensity, hue, saturation and fusion work.

Fusion rules are critical factors of image fusion, and different fusion rules are required for different purposes during image fusion. SARs can image under various environmental conditions, but the interpretation of SAR images is difficult. It is the object of this study to improve the interpretability of SAR images by injecting the information of multispectral images into SAR images, so that people can quickly get and interpret information of the scenes concerned at any time instead of the image fusion mentioned in the previous literature and to integrate more information to improve people’s understanding of the research area. Therefore, the SAR image information should be maintained as much as possible in the fusion rules. After the images are decomposed through multi-resolution analysis method, the low-frequency components represent the background or spectral information of the images, which is the common information of a large area in the images. For example, the color of vegetation in an optical image is green, but the color of water in its SAR image is black. Therefore, for the fusion of low-frequency components, this paper suggests that the spectral information of multispectral images be injected in a certain proportion to improve the background interpretation of SAR images. Here, the correlation coefficients of the two kinds of images are used as weights for the fusion, as is shown in Equation (3) [4].

(3) $$LF_{new}=w_{1}\times LF_{I}+w_{2}\times LF_{SAR}$$

(4) $$w_{1}=\mathrm{corr}(LF_{SAR},LF_{I})=\frac{\sum \left(LF_{SAR}-\bar{LF_{SAR}})(LF_{I}-\bar{LF_{I}}\right)}{\sqrt{\sum {\left(LF_{SAR}-\bar{LF_{SAR}}\right)}^{2}\sum {\left(LF_{I}-\bar{LF_{I}}\right)}^{2}}}$$

(5) $$w_{2}=1-w_{1}$$

where $LF_{SAR}$, $LF_{I}$ are the low frequent components of intensity component and SAR image decomposed using non-subsampled shearlet transform, respectively. $\bar{LF_{SAR}}$ and $\bar{LF_{I}}$ are average values of $LF_{I}$, $LF_{SAR}$. $w_{1}$ is the correlation coefficient between the low frequent components of intensity component and SAR image. $w_{1}$+$w_{2}$ = 1, $w_{1}$ and $w_{2}$ are used as weight value of low frequent component combination.

The high-frequency coefficients reflect their detail spatial information. The greater the change of the high-frequency coefficients is, the richer the detail information will be. The high-frequency coefficients of the fused images can be determined according to the absolute value of the pixel, the energy of the region or the energy of the object. In this paper, the improved activity measure of regional energy [29] is used to select the coefficients, which is defined as Equations (6)–(8),

(6) $$A_{I}=\sum_{}W(s,t)\left|D_{I}\left(i+s,j+t\right)\right|$$

(7) $$A_{\mathrm{SAR}}=\sum W(s,t)\left|D_{SAR}\left(i+s,j+t\right)\right|$$

(8) $$D_{\mathrm{new}}=\{\begin{array}{c} D_{\mathrm{SAR}}\;A_{\mathrm{SAR}}>A_{I}\\ D_{I}\;\;else\;\;\; \end{array}$$

where, $D_{\mathrm{SAR}}$ and $D_{I}\;$ are the high frequent coefficient of the intensity component and SAR image, respectively. $A_{I}$ and $A_{\mathrm{SAR}}$ are the activity measure of the high frequent coefficient, respectively. W(s,t) is a coefficient matrix.

(9) $$W(s,t)=\left|\begin{array}{ccc} 1/16 & 1/16 & 1/16\\ 1/16 & 1/2 & 1/16\\ 1/16 & 1/16 & 1/16 \end{array}\right|$$

Compared with the method proposed by Hong et al. [4] and Chu et al. [15], the method proposed here looks similar, but there are many differences in details. According to Hong’s method, wavelet transform is used to decompose the images. In the description of image details, it is difficult to describe entities in different directions through wavelet transform compared with shearlet transform. In terms of the fusion of high-frequency coefficients, Hong et al. directly apply the high-frequency coefficients of SAR images. Although the SAR image information is completely retained through this fusion, the spectral information of optical images is injected, and the interpretation ability of SAR images is improved, there is no detail spatial information injection of optical images. The details of SAR images are a response of electromagnetic backscattering, which is not easy to be interpreted by humans. If there is a background of visible image details, the interpretation of SAR image details will be easier. According to the method of Chu et al. [15], the coefficient fusion in the low-frequency domain is to calculate the weight according to the regional variance to fuse the images, so that the fusion coefficients of each pixel of the images are different. High-frequency threshold coefficient fusion is also selected according to the regional energy of the coefficients. The advantage of this fusion method is that the outstanding information of the original images is integrated as much as possible, but the disadvantage is that the original information of SAR images is completely destroyed in the fusion process, resulting in a complete loss of SAR image information in a pixel or region of the fused images, which cannot meet the purpose of improving the interpretation ability of SAR images.

### 2.4. Evaluation Metric

In order to evaluate the performance of the method proposed in this paper, both subjective visual evaluation and several objective indexes are employed. These indexes include correlation coefficients (CC) [29,30], high-frequency correlation coefficients (HFCC) [31], the average gradient (AG) and the information entropy (IE). Among them, CCs are used to evaluate the spectral similarity between the fused images and the source images. The larger the correlation coefficients are, the more spectral information in source images will be integrated into SAR images, as is given in Equation (4). HFCCs between SAR images and fused images show the degree of spatial details of SAR images injected into the fused images, whose idea is to extract the high-frequency information of the images through a high-frequency filter, and then calculate the correlation coefficients of the high-frequency information. The high-frequency filter shown in Equation (10) is used here. The average gradient of the images reflects their clarity. The greater the average gradient is, the clearer the images are, indicating that more spatial details are fused into the images, as is given in Equation (11).

(10) $$\left|\begin{array}{ccc} -1 & -1 & -1\\ -1 & 8 & -1\\ -1 & -1 & -1 \end{array}\right|$$

(11) $$\nabla \overset{-}{G}=\frac{1}{\mathrm{MN}}\sum_{i=1}^{M}\sum_{j=1}^{N}\sqrt{\Delta xF^{2}(x,y)+\Delta yF^{2}(x,y)}$$

where $\nabla \overset{-}{G}$ is the average gradient of image,ΔxF(x,y), ΔyF(x,y) are the difference of the image along the X and Y directions respectively.

The information entropy of the images reflects the average amount of information in the images and represents the amount of information in the injected fused images. The larger the information entropy is, the richer the information will be in the fused images, as is calculated through Equation (12).

(12) $$EN=-\sum_{i=0}^{L-1}p_{i}\log_{2}p_{i}$$

where L is the range of image gray value, $p_{i}$ is the probability of occurrence of $i^{th}$ gray level.

### 2.5. Comparison Methods

Three typical fusion methods are chosen to compare with the method proposed in this paper, namely IHS transform fusion method [12], DWT-HIS fusion method [4] and contourlet-IHS fusion method. According to the DWT-HIS method, contourlet-IHS method and the method proposed in this paper, the level of multi-resolution decomposition is three. The wavelet basis of the DWT-IHS fusion method is DB2 filter. The fusion rule of the contourlet-IHS fusion method is the same as that of the DWT-HIS method.

## 3. Results and Analysis

### 3.1. Results

**Figure 3:**
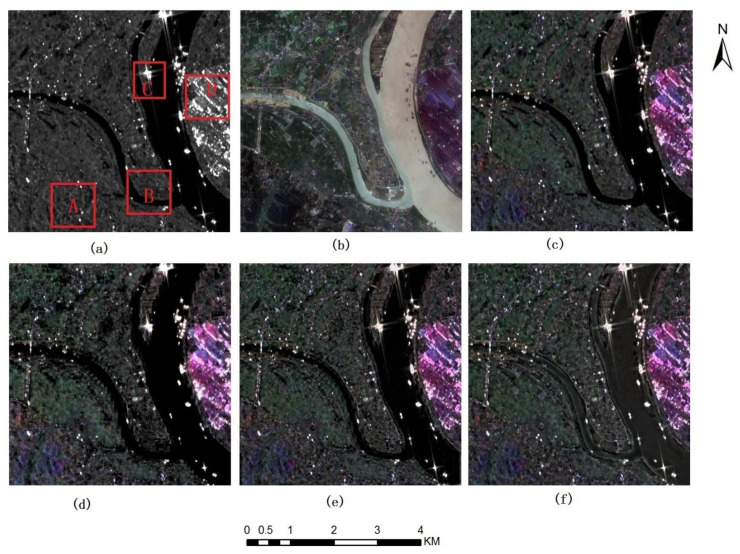
Sentinel 1A SAR VV polarization image and Sentinel 2A multispectral images near Xiangtan City, China’s Hunan Province and fusion images of different method (**a**) Sentinel1A VV image (**b**) sentinel2A Band432 images (**c**) fusion images of IHS (**d**) fusion images of DWT-IHS (**e**) fusion images of NSCT-IHS (**f**) fusion images of the method proposed.

**Figure 4:**
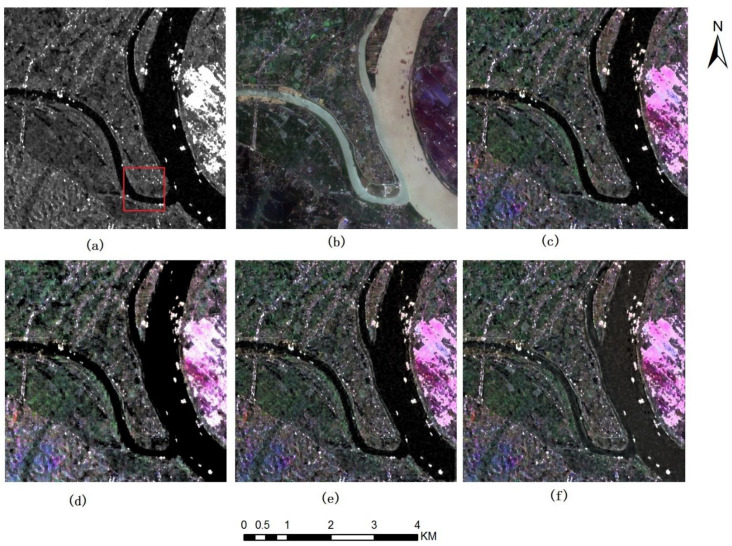
Sentinel 1A SAR VH polarization image and Sentinel 2A multispectral images near Xiangtan City, China’s Hunan Province and fusion images of different method (**a**) Sentinel1A VH image (**b**) sentinel2A B432 images (**c**) fusion image of IHS (**d**) fusion image of DWT-IHS (**e**) fusion image of NSCT-IHS (**f**) fusion image of the method proposed.

Figure 3 and Figure 4 show the fusion results of Sentinel 1A VV SAR images, VH SAR images and Sentinel 2 A multispectral images (Band 4, 3 and 2, namely true color images). Among them, Figure 3a and Figure 4a show Sentinel 1A VV and VH SAR images respectively, Figure 3b and Figure 4b show Sentinel 2 multispectral images, Figure 3c–f and Figure 4c–f show the fused images based on IHS, DWT-IHS, NSCT-IHS as well as the method proposed in this paper, respectively.

**Figure 5:**
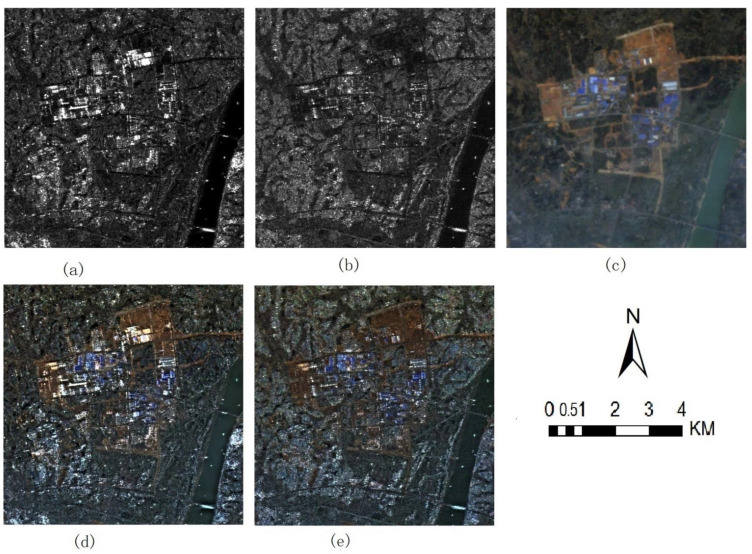
ALOS SAR and Landsat5 TM multispectral images and fusion images using the method proposed in this paper (**a**) ALOS HH image (**b**) ALOS HV image (**c**) landsat5-321 images (**d**) fusion image of ALOS HH image and landsat5-321 images (**e**) fusion image of ALOS HV image and landsat5-321 images.

**Figure 6:**
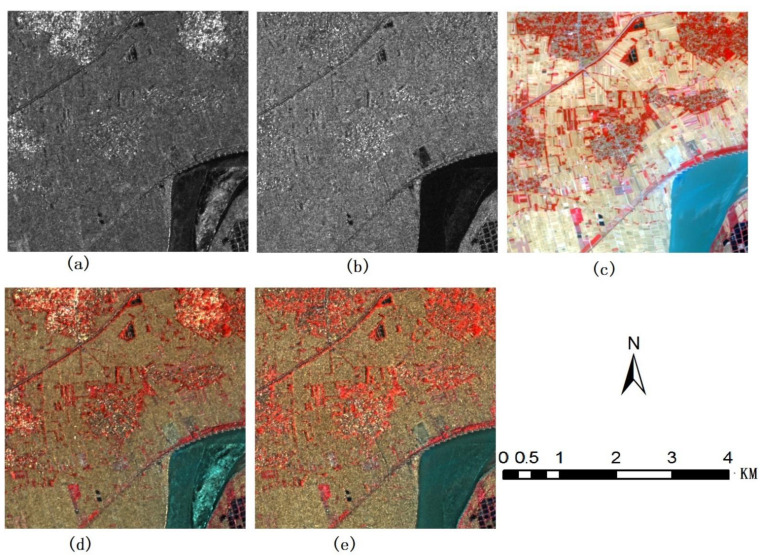
GF3 SAR HH image, GF3 SAR HV image and senbinel2A multispectral images near Xiangtan City, China’s Hunan Province and fusion images using the method proposed in this paper (**a**) GF3 HH image (**b**) GF3 HV image (**c**) Sentinel 2A-843 images (**d**) fusion image of GF3 image and Sentinel 2A-843 images (**e**) fusion image of GF3 HV image and Sentinel 2A-843 images.

Figure 5 shows ALOS HH and HV SAR images, original Landsat TM images (Band 3, 2 and 1), and fused images of the Landsat TM with ALOS SAR data using the proposed method. Figure 6 shows GF3 HH and HV SAR images, original Sentinel 2 images (band 8, 4 and 3) and fused images of the Sentinel 2 multispectral images with GF3 SAR data using the proposed method.

### 3.2. Visual Analysis

Figure 3a, Figure 4a and Figure 5a,b as well as Figure 6a,b show SAR images of different SAR sensors in different polarization modes. From the figures, we can find that there are great differences among these SAR images in different polarization modes. Figure 3a shows the images in VV polarization mode. Compared with Figure 4a showing VH polarization mode, the white bright spots contained in it are denser and clearer. By comparing with multispectral images Figure 3b, it is found that these white highlights correspond to the metal ceiling of the building. At the same time, from Figure 5a,b and Figure 6a,b, we can find that:

The building areas in Figure 5a and Figure 6a are easy to distinguish, whose polarization mode is HH. Considering Figure 3a, it shows that HH or VV SAR polarization imaging mode is more suitable for the study of building areas. However, if we observe SAR images alone, we can only roughly judge the outline of the features of the images, and it is difficult to identify the types of features. But in fused images, such as Figure 3c–f, the spectral information of multispectral images is integrated into SAR images, and most information of ground objects is easier to be interpreted, so that we can identify buildings, water bodies, vegetation and other ground features. There is a strong scattering feature in Region C of Figure 3a, considering the combination with Figure 3b, the corresponding area reflects buildings. Buildings with strong scattering information are obtained in the fused images, and the complementary information of SAR and multispectral images is also effectively integrated.

Figure 3c–f are fused images of IHS, wavelet-IHS, contourlet-IHS and the method proposed respectively, and source images are Sentinel 1A VV SAR and Sentinel 2A multispectral images. Compared with the source multispectral images, the colors of all the fused images look close to those of the multispectral images. However, there are still serious spectral distortions in some regions, such as Region A, Region D and waters in Figure 3a. In terms of the degree of color integration in multispectral images, the fused images through the proposed method are visually closer to the original multispectral images than those through the other three methods.

Region B in Figure 3a includes roads, water bodies, bare land and vegetation, which are difficult to distinguish in the image in Figure 3a. In the fused images (Figure 3c–f), the clarity of the road is gradually improved. In Figure 3f, the road is the clearest and can be easily recognized. In Figure 3c–e, the water bodies are very similar, showing black, but in Figure 3f, the water bodies contain some light tone areas. The bare land area is basically unrecognizable in Figure 3c,d, and the distribution range can only be roughly identified in Figure 3e,f. The vegetation area can be well identified in Figure 3e,f, but Figure 3f can better show the color depth change of the vegetation and the boundary of the vegetation area. From the details of the fusion image, the method used in this paper can better capture the detailed features of the ground objects and fuse them.

According above all, the description of ground objects in the fused images produced through the method proposed in this paper is obviously more delicate than that through other methods, and it is easier for people to interpret the types and detailed features of ground objects.

Figure 4 shows the fused image of Sentinel 1A VH SAR image and Sentinel 2A multispectral image based on different fusion methods. From this group of fused images, we can also find that the spectra of the fused images are closer to those of the multispectral images, which is more obvious than that of the dataset in Figure 3. From an enlarged view of the red rectangular area in Figure 4a. It can be seen from the figure that the fused images through the method proposed in this paper contain more color and detail information than other images, which people can recognize more easily, such as roads and roadside farmland with different colors. It is worth noting that there is a white spot in the red area of Figure 4a. We cannot determine which kind of ground object it is, but the fusion image clearly shows that the spot is in a building area beside the road, which makes it easy to judge that the spot is a house with a metal roof.

In Figure 5, an ALOS HH SAR image and an ALOS HV SAR image are fused with Landsat 5 TM images (Band 3, 2 and 1) using the method proposed in this paper. The electromagnetic frequency of ALOS SARs is L-band, and its penetration ability to the earth surface is stronger than that of C-band SARs, making it easier to reflect the characteristics of ground objects under the surface. Figure 5d,e show the fused images using the method proposed, Figure 5d is the fusion image of ALOS HH image and landsat5-321 images, Figure 5e is the fusion image of ALOS HV image and landsat5-321 images. The colors of the fused images are very close to those of the Landsat multispectral images. In fusion images, different types of buildings and common land types can be easily distinguished, such as water, vegetation and bare land, etc. In terms of interpretation ability, the fused images are significantly stronger than the original SAR images, based on which

Figure 6 shows the third dataset. GF3 HH SAR images and GF3 HV SAR images are fused with a Sentinel 2 multispectral image (Band 8, 4 and 3) using the method in this paper. The spatial resolution of SAR images is 8.5 m, while that of the multispectral image is 10m. The bands of the multispectral image include near-infrared, red- and green-light bands. Thus, the color of the vegetation is red. The color of the fused image, on the whole, is closer to that of the multispectral image, but the details of the image are much richer, people can easily distinguish vegetation in the image, which is a difficult task to achieve in the original SAR images because of the colors injected from Sentinel 2 multispectral images. Compared with the original SAR images, the interpretation ability of the fused image has been greatly improved.

### 3.3. Statistical Analysis

The above content is the result of a subjective evaluation. In order to evaluate the effectiveness of this method more comprehensively, four objective evaluation indicators, namely correlation coefficients, high-frequency-component correlation coefficients, average gradient and information entropy are used for the quantitative comparative analysis in this paper. For multi-band images, the calculated parameters take the average value of all band images.

Table 2 lists the mean correlation coefficients (MCCs) and mean high-frequency-component correlation coefficients (MHFCCs) in the fused images (Sentinel 2A Band 3, 2 and 1 and Sentinel 1A VV SAR) and the Sentinel 1A VV SAR images. Table 2 shows that the MCCs of the original multispectral images and VV-polarization-mode SAR images are -0.364, which means that the spectral correlation between the two kinds of images is relatively weak, and the gray value distribution even has an opposite trend. At the same time, the high-frequency correlation coefficients are only 0.047, which indicates that the spatial details (namely high-frequency component) of the two kinds of images are basically irrelevant. This is mainly due to the difference in the imaging principles of images. SAR images reflect the backscattering characteristics of ground objects on C-band electromagnetic waves, while multispectral images reflect the reflection characteristics of ground objects on visible light bands. It can be seen that all the MCCs and MHFCCs of the fused images and the SAR images have been greatly improved compared with the mean correlation coefficients of original images, which indicates that the information of SAR images is well integrated into the fused images. Among these indexes, the IHS method has the highest index, which is caused by the fact that the intensity components are completely replaced by the SAR images in the fusion process, and there is no loss of SAR information. This also causes a result that the information of multispectral images is injected less than that through other methods, and the interpretation ability of fused images is weaker than that through other methods. The MHFCC of the fused images using the method proposed in this paper is 0.706, which is the largest except through the IHS method. It shows that, compared with the fusion method based on multi-scale analysis, this method is better than wavelet transform and contourlet transform in integrating the spatial details of SAR images. The MCC of the proposed Shearlet-IHS method is 0.853, which is less than that of the other three methods, indicating that this method is weaker than other methods in the integration of spectral information of SAR images. Of course, this index is not very important, because the gray value of SAR image reflects the scattering characteristics of ground objects on electromagnetic waves, which is not very helpful for image interpretation. Table 3 lists the mean correlation coefficients (MCCs) and mean high-frequency-component correlation coefficients (MHFCCs) of the fused images (Sentinel 2A Band 3, 2 and 1 and Sentinel 1A VH SAR) and the Sentinel 1A VH SAR images. The change trend of these coefficients is similar to that in Table 2.

Table 4 lists the MCCs and MHFCCs of the fused images (Sentinel 1A VV SAR and Sentinel2A multispectral images) and the Sentinel 2A multispectral images. It can be seen that from the IHS method to the method proposed here, the correlation coefficients of the fused images and the multispectral images change greatly, from −0.351 to 0.004, indicating that the multispectral image information integrated in the fused images is gradually increasing, but the value is not very large. Compared with the multispectral images, there is a serious spectral distortion, which is mainly caused by the different imaging principles of the images. Compared with other methods, the MHFCCs through the proposed method in this paper are the largest, which means that the details of multispectral images can be better injected into the fused images through this method. Because the correlation coefficient and high-frequency correlation coefficient of the method proposed are the largest, it shows that the method proposed can better integrate the spectral information and spatial information of multispectral images into the fusion image than other methods, and improve the interpretation ability of the fusion images. Table 5 lists the MCCs and MHFCCs of the fused images (Sentinel 1A VH SAR and Sentinel 2A multispectral images) and the Sentinel 2A multispectral images. The change trend of these coefficients is similar to that in Table 4.

Table 6 lists the gradient and information entropy of Sentinel 1A VV SAR images and the fused images based on different methods. It can be seen from the table that compared with the SAR images before fusion, the gradient and information entropy of the fused images have increased. This means that the fused images have a better clarity and richer information than the original SAR images. Among them, the value of contourlet-IHS and the method proposed here has obtained maximum value, and the difference between them is very small. This shows that there is no obvious difference between the method proposed and contourlet-IHS method in this aspect, and the best value is obtained in image clarity and information amount. Table 7 lists the gradient and information entropy of Sentinel 1A VH SAR images and fused images based on different methods. The change trend of these coefficients is similar to that in Table 6.

In order to evaluate the applicability of the proposed algorithms more comprehensively, Table 8 lists the correlation coefficients and high-frequency-component correlation coefficients of the fused images and the original images generated through the method in this paper for the other two datasets. The first and second line are the correlation coefficients and high-frequency-correlation coefficients of the original images and fused images, which are fused using ALOS HH/HV SAR images and Landsat 5 TM images. The third and fourth line are the correlation coefficients and high-frequency correlation coefficients of the original images and fused images, which are fused using GF3 HH/HV SAR images and Sentinel 2A Band 8, 4 and 3 images.

It can be seen that the correlation coefficients between fused images and SAR images increase significantly compared with those between SAR images and original multispectral images, especially for ALOS and Landsat 5 TM images, the correlation coefficients reach 93%, which shows that multispectral image information can be integrated into SAR images well through this method. At the same time, the high-frequency correlation coefficients between SAR images and fused images are much larger than those between SAR images and original multispectral images, which shows that the details of SAR images are also well preserved in the fused images.

## 4. Discussion

Among various image fusion algorithms, the hybrid method combining multi-scale decomposition method with IHS method is one of the most popular methods used by researchers. Theoretically, the more levels and directions of image decomposition are, the more details of the images will be described, and the better the image fusion effect will be. Because different polarization modes will cause SAR images to show different characteristics, the effect of different fusion methods on the fusion of SAR images with different polarization modes and multispectral images is rarely mentioned in previous studies. Therefore, new multi-scale decomposition tools are used for the method proposed in this paper, to fuse SAR images, namely shearlet transform and IHS transform, and their fusion effect is compared with that of traditional wavelet transform and contourlet transform. In this paper, Sentinel 1A VV SAR image data, Sentinel 1A VH SAR and Sentinel 2A multispectral data are selected for experiments. The experimental results indicate that the visual interpretation effect and statistical indexes of the two groups of experimental data through the method proposed are better than that of other methods, which shows that this method is better than other traditional methods in fusing SAR images and multispectral images as well as improving SAR image interpretation ability. C-band GF3 SAR images, L-band ALOS radar data and multispectral image fusion are also selected in this paper. The fusion results also show that good results have been achieved through this method in L-band and C-band SAR image fusion.

However, although good results have been achieved through the method proposed in the fusion of SAR and multispectral images with different electromagnetic frequencies and spatial resolutions, there is still a problem to be further studied in the multi-resolution decomposition and fusion algorithms, that is, the determination of the optimal decomposition scale of the images. Due to different content of the images, the number of layers and directions of image decomposition should change automatically in the fusion process, but these parameters are fixed at present, which may cause excessive or insufficient image decomposition, and the image fusion effect cannot be optimized. This problem needs to be further studied in the future.

## 5. Conclusions

The interpretation of SAR images is the basis of SAR image application, but due to its imaging mechanism, it is very difficult to interpret SAR images directly. Fusing them with multispectral images is one of the effective means to improve the interpretation ability of SAR images. On the basis of previous research, a fusion method combining shearlet transform with activity measure is proposed in this paper, whose aim is to improve the interpretation ability of SAR images, rather than to simply improve the amount of information in the fused images. In order to evaluate the effectiveness of this method, SAR images from three different sensors, Sentinel 1A, ALOS and GF3, are selected here. These SAR working electromagnetic bands are L-bands and C-bands respectively. The polarization modes of the SAR images include HH, VV, HV and VH. The applied multispectral images contain Sentinel 2 and Landsat 5 TM images, and the designed bands include blue, green, red and near-infrared bands. The fused images through the method proposed are visually compared and statistically analyzed based on the fusion results through previous IHS methods, DWT-IHS methods and contourlet-IHS methods. The description of ground objects in the fusion image produced by the method proposed is obviously more delicate than other methods visually. In the fusion of sentinel 1A VV image and sentinel 2A multispectral images, the mean high-frequency correlation coefficient between the fusion images generated by the method proposed and the SAR images is 0.706, and the mean correlation coefficient with the multispectral images is 0.004, which are higher than the indexes of the other two hybrid methods. The high-frequency correlation coefficient between the fusion image of IHS method and SAR image is the largest, but the correlation coefficient with multispectral images is the smallest. Although IHS method can integrate the spatial information of SAR image into the fusion image well, its ability is the weakest in the fusion of spectral information. The results of other groups of tests are similar to the above results. All these show that through the method proposed in this paper, the spectral information can be better integrated with the spatial detail information of multispectral images while maintaining the spatial information of the original SAR images, meanwhile effectively improving the interpretation ability of SAR images.

## Figures and Tables

**Table 1:** Sensor spectral range and resolution.

Sensor	Spatial Resolution (m)	Spectral Range (µm)	Polarization Model
Sentinel 1A	15	C-band	VV, VH
ALOS PALSAR	12.5	L-band	HH, HV
GF3	8.5	C Band	HH, HV
Sentinel 2A (band2-4,8)	10	0.458–0.523, 0.543–0.578, 0.65–0.68, 0.785–0.9	
Landsat5 TM (band1-3)	30	0.4–0.52, 0.52–0.60, 0.63–0.69	

**Table 2:** Mean correlation coefficients and mean high frequency component correlation coefficients between the fusion image and the Sentinel 1A VV SAR image.

		CC	HFCC
Sentinel2	R	−0.341	0.051
	G	−0.419	0.039
	B	−0.337	0.051
	Mean	−0.365	0.047
HIS	R	0.988	0.936
	G	0.958	0.903
	B	0.987	0.912
	Mean	0.977	0.917
DWT+HIS	R	0.909	0.636
	G	0.896	0.620
	B	0.902	0.602
	Mean	0.902	0.619
Contourlet+HIS	R	0.937	0.712
	G	0.890	0.644
	B	0.934	0.678
	Mean	0.920	0.678
Shearlet+HIS	R	0.884	0.744
	G	0.828	0.670
	B	0.891	0.706
	Mean	0.853	0.706

**Table 3:** Mean correlation coefficients and mean high frequency component correlation coefficients between the fusion image and the Sentinel 1A VH SAR image.

	MCC	MHFCC
Sentinel2	−0.426	0.048
IHS	0.977	0.932
DWT-IHS	0.928	0.716
Contourlet -IHS	0.949	0.815
The method proposed	0.928	0.850

**Table 4:** Mean correlation coefficients and mean high frequency component correlation coefficients between the fusion images (sentinel1A VV and Sentinel2A Band 432) and the Sentinel 2A multispectral images.

	MCC	MHFCC
IHS	−0.351	0.133
DWT−IHS	−0.315	0.553
Contourlet−IHS	−0.238	0.620
The method proposed	0.004	0.677

**Table 5:** Mean correlation coefficients and mean high frequency component correlation coefficients between the fusion images (sentinel1A VH and Sentinel2A Band 432) and the Sentinel 2A multispectral images.

	MCC	MHFCC
IHS	−0.422	0.127
DWT−IHS	−0.455	0.425
Contourlet −IHS	−0.353	0.432
The method proposed	−0.173	0.446

**Table 6:** The gradient and information entropy of Sentinel 1A VV SAR image, Sentinel 2A multispectral images, and fusion image based on different methods.

	Gradient	Information Entropy
Sentinel1 VV	10.082	6.154
IHS	10.976	6.240
DWT-IHS	14.847	6.242
Contourlet -IHS	15.380	6.586
The method proposed	15.284	6.580

**Table 7:** The gradient and information entropy of Sentinel 1A VH SAR image, Sentinel 2A multispectral images, and fusion image based on different methods.

	Gradient	Information Entropy
Sentinel1 VH	16.188	6.014
IHS	17.178	6.694
DWT-IHS	19.284	6.462
Contourlet -IHS	20.066	7.034
The method proposed	19.866	6.958

**Table 8:** Mean correlation coefficients and mean high frequency component correlation coefficients between original images and fusion images using the proposed method.

	MCC		MHFCC	
	SAR with MS	Fusion Image with MS	SAR with MS	Fusion Image with SAR
ALOS HH SAR with Landsat 5 TM	0.112	0.931	0.063	0.988
ALOS HV SAR with Landsat 5 TM	−0.097	0.939	0.087	0.988
GF3 HH with sentinel 2	−0.015	0.614	0.121	0.740
GF3 HV with sentinel 2	0.166	0.552	0.122	0.775

## Data Availability

The Sentinel 1A and ALOS SAR data from the ASF DAAC can be obtained at https://asf.alaska.edu/#/ (accessed on 5 March 2022). The Sentinel 2 and Landsat 5 data from USGS are available at https://earthexplorer.usgs.gov/ (accessed on 7 March 2022).
